# Cave-dwelling bats as hosts of *Trypanosoma livingstonei*: Evidence from peri-domestic roosts in southeastern Nigeria

**DOI:** 10.1371/journal.pone.0355840

**Published:** 2026-08-12

**Authors:** Elijah Sunday Okwuonu, Henry Ekene Nnabuife, Benneth Chigozie Obitte, Oskar Werb, Iroro Tanshi, Tigga Kingston, Ikem Chris Okoye, Joshua Kamani, Juliane Schaer

**Affiliations:** 1 Department of Zoology and Environmental Biology, University of Nigeria, Nsukka, Enugu State, Nigeria; 2 Parasitology Division, National Veterinary Research Institute (NVRI) Vom, Plateau State, Nigeria; 3 Small Mammal Conservation Organization (SMACON), Benin City, Edo State, Nigeria; 4 Department of Biological Sciences, Texas Tech University, Lubbock, Texas, United States of America; 5 Department of Molecular Parasitology, Institute of Biology, Humboldt University of Berlin, Berlin, Germany; 6 Department of Biology, University of Washington, Seattle, Washington, United States of America; 7 Department of Evolution, Ecology and Behavior, School of Integrative Biology, University of Illinois, Urbana, Illinois, United States of America; 8 Department of Biology, Muni University, Arua, Uganda; 9 Department of Biological Sciences, Macquarie University, Sydney, New South Wales, Australia; University of Ibadan Faculty of Veterinary Medicine, NIGERIA

## Abstract

Bats play important roles in ecosystem functioning and host a remarkable diversity of blood parasites, including a limited number of bat-associated *Trypanosoma* species that have been described from Africa. Cave roosts provide critical habitat for bats and often support large, multispecies aggregations that may influence parasite transmission dynamics. Nevertheless, the diversity and host associations of trypanosomes within these environments remain poorly characterized, particularly in West Africa. To address this gap, we screened blood and ectoparasite samples from 57 bats representing ten species captured in nine caves located 0.4–2 km from human residences, as well as from one residential building in southeastern Nigeria, using PCR. Trypanosome DNA was detected in 22/57 bats (38.6%) and in six bat species across four families: *Rousettus aegyptiacus* (1/2), *Hipposideros abae* (11/23), *H. jonesi* (1/1), *H. ruber* (5/11), *Rhinolophus landeri* (2/10), and *Mops condylurus* (2/6). Phylogenetic analyses placed the trypanosome sequences within the *Trypanosoma livingstonei* species group, a clade of African bat-associated trypanosomes representing early-diverging lineages within the *Trypanosoma cruzi* clade, which includes the causative agent of Chagas disease. The Nigerian trypanosome sequences were not recovered as a single homogeneous lineage, and species delimitation analysis recovered them together with other *T. livingstonei/ T.* cf. *livingstonei* references across several putative molecular lineages. The exclusive detection of parasites of the *T. livingstonei* species group across multiple bat species indicates low trypanosome diversity within this cave system but reveals phylogenetic structuring within this parasite group. The findings highlight the need for broader geographic sampling, multilocus data, and ecological measurements to clarify the drivers of trypanosome diversity in cave-dwelling bats.

## Introduction

Trypanosomes are protozoan parasites of medical and veterinary importance [1]. Members of the genus *Trypanosoma* exhibit remarkable genetic diversity in bats, with several lineages closely related to species infecting other mammals, including livestock and humans [2–6]. Phylogenetic evidence further supports bats as ancestral hosts in the evolutionary history of the *Trypanosoma cruzi* clade*,* the group that includes the agent of Chagas disease [7]. Although Chagas disease occurs primarily in rural areas of Mexico, Central and South America, multiple lineages within the *T. cruzi* clade, including *Trypanosoma livingstonei*, are thought to have originated in African bats [5,7,8]. This evolutionary context highlights the importance of systematic sampling and molecular characterization of trypanosomes in African bat communities. Since its first description in 2013 from two bat species in Mozambique*, T. livingstonei* has been reported from several bat species across Africa [6,8–10]. However, its host associations, patterns of occurrence within multispecies assemblages, and potential ecological drivers remain insufficiently understood. The potential for interspecific transmission is likely enhanced in bat species that roost together in stable environments such as caves. Cave systems provide constant microclimatic conditions, high host densities, and repeated interspecific contact, all of which may influence parasite dynamics. Expanding research on bat trypanosomes in understudied regions, including southeastern Nigeria, is therefore important for clarifying host–parasite associations and understanding how ecological context shapes parasite distribution [6,7,11].

Bats also host a remarkable diversity of ectoparasites, reflecting their wide geographic distribution, social behavior, and ecological versatility [12,13]. Bat flies (Nycteribiidae and Streblidae), mites (Spinturnicidae), fleas (Ischnopsyllidae), and ticks (Argasidae) are common and often form intimate, co-evolved associations with their hosts [14,15]. These ectoparasites form close associations with their hosts and represent potential components of bat-associated parasite systems.

In sub-Saharan Africa, cave-dwelling bats represent a particularly suitable system for studying host-parasite interactions. Enugu State in southeastern Nigeria, characterized by extensive sandstone cave systems, supports diverse bat assemblages. Despite Nigeria supporting a high bat species richness of approximately 100 bat species [16,17], protozoan blood parasites of cave-roosting bats remain poorly studied. While investigations on bat trypanosomes have been conducted in parts of West Africa, including the arid lands of northern Nigeria, comparable studies are lacking for the wet tropical ecosystems of southern Nigeria, where bat species richness is among the highest on the continent [16,18,19].

Here, we investigate the presence and diversity of trypanosomes and associated ectoparasites in cave-dwelling bats from southeastern Nigeria. Using molecular approaches, we aim to characterize parasite diversity, assess host associations across multispecies cave assemblages, and examine their phylogenetic relationships within the broader context of African bat-associated trypanosomes.

## Materials and methods

### Study area

**Fig 1:**
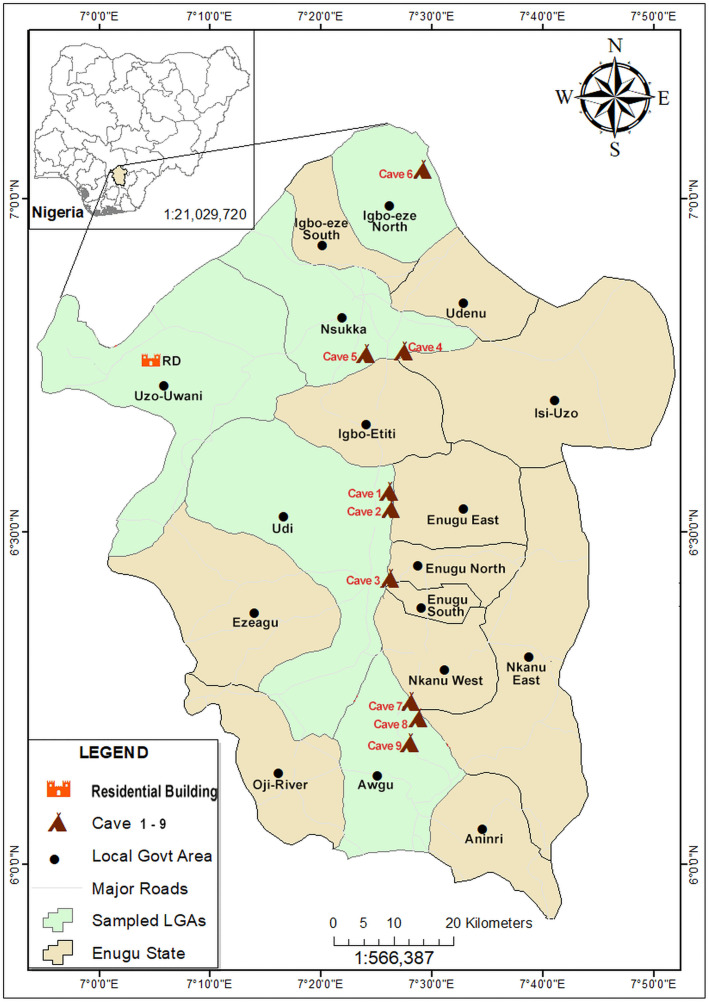
Map of Enugu State, southeastern Nigeria. Locations of nine cave roosts and one residential building where bats were sampled are indicated. Sampling sites are marked with symbols, as shown in the legend. LGA = Local Government Area. Map created by Chukwu, Matthew Tochukwu, Cpraise Evaluations and Geospatial Aid (CEVGEO™), reproduced under the Creative Commons Attribution 4.0 International License (CC BY 4.0).

The study was conducted in nine caves and one residential building used as bat roosts across eight communities in Enugu State, southeastern Nigeria (Fig 1). Enugu State is situated on the Udi-Nsukka Plateau, whose geology comprises crystalline basement rocks with localized sedimentary cover and extensive sandstone formations. These geological features create a fractured, relief-rich landscape supporting numerous cave systems.

Caves occur along escarpments, river valleys, and fault corridors and include both natural formations and anthropogenic voids (e.g., abandoned buildings, quarry pits, and building crevices). Peri-domestic roosts, caves and cave-like structures near settlements, were prioritized to characterize cave-dwelling bat communities and assess the presence of *Trypanosoma* parasites.

Caves in the region are used for a variety of purposes, including recreation and exploration, educational and cultural activities led by schools and guides, spiritual or seasonal practices, and as places for shelter or temporary storage. They also support local economies and protein needs through tourism and hunting. Visit frequency includes regular local use, occasional visits, seasonal trends, and events, which are shaped by factors such as trail ease, safety considerations, and the availability of managed paths.

### Ethics statement

Ethical approval for this study was obtained from the Ethics and Biosafety Committee, Faculty of Biological Sciences, University of Nigeria, Nsukka (approval number: UNN/FBS/EC/1014). Fieldwork and sample collection were conducted with permission from the relevant institutional and regulatory authorities in Nigeria, including the State Ministries of Health and Environment. Where required, access to sampling sites was granted with the approval and support of local community leadership.

All bat capture, handling, and sampling procedures followed the guidelines of the American Society of Mammalogists for the use of wild mammals in research [20], and all efforts were made to minimize stress and harm to the animals.

Extracted DNA samples were transferred to Germany in 2024 for molecular analysis under a formal Material Transfer Agreement (MTA) between the University of Nigeria, Nsukka, and Humboldt University of Berlin (signed 16 July 2024). The transferred material consisted solely of extracted DNA and not whole biological specimens. The samples were derived from bat species that are not listed as threatened, and to our knowledge, no specific export or import permits were required for this type of material. The extracted DNA was fully utilized during the analyses, and no material remains for return or further transfer.

### Sampling (field methods)

The study is part of a broader investigation of cave-dwelling bat ecology in Enugu State (S1 Table). Sampling was conducted between 2020 and 2022 (January-February 2020, July-August 2021, and February-September 2022).

Caves were selected based on proximity to human settlements, evidence of bat activity, and permission granted by community leaders. The nine selected caves were located approximately 0.4–2 km (5–30 minutes walking distance, depending on terrain) from human residences. Bats were captured using harp traps and ground-level mist nets of varying lengths (6 m, 9 m, and 12 m) placed at cave entrances [21]. Triple-high mist nets were deployed at the residential building. Harp traps were primarily used for insectivorous bat species, whereas mist nets were used for the fruit bat species *Rousettus aegyptiacus*. Vegetation was strategically arranged to reduce exit routes and improve capture efficiency [22]. Nets were opened at sunset (6:00–8:00 PM) and monitored continuously until approximately 20 individuals were captured, after which nets were closed. Sampling was conducted over two consecutive nights per site.

Captured bats were placed individually in breathable cloth bags and identified to species level using morphological identification keys [23,24]. A 3 mm biopsy punch was used to collect wing tissue for identification of recaptured individuals. Ectoparasites were collected with fine forceps and brushes and preserved in absolute ethanol. Blood samples were obtained via venipuncture of the cephalic vein [25]. Blood volumes did not exceed 0.6–1% of body mass, remaining within established safety margins relative to estimated total blood volume [20]. To minimize discomfort and prevent infection, a lignocaine–amoxicillin paste was applied to the puncture site. Whole blood was stored in EDTA tubes. All procedures were performed by trained personnel. Most bats were released at the site of capture immediately after sampling. A subset of individuals (14/57) was retained as voucher specimens; these individuals were euthanized using isoflurane and preserved in absolute ethanol and are deposited in the SMACON (the Afrotropical Biodiversity Center) museum collection, where they retain their original specimen identification numbers (S1 Table). The use of isoflurane followed established guidelines for humane euthanasia of small mammals [20]. All remaining individuals were released at the sampling site following recovery. Fruit bats (Pteropodidae) were provided with a sugar solution prior to release, whereas no supplemental feeding was provided to insectivorous species.

### Microscopy and ectoparasite identification

Thin blood smears were prepared, air-dried, and fixed in absolute methanol for 60 seconds. Slides were then stained with Giemsa for 45 minutes, gently rinsed with phosphate-buffered saline (PBS), and allowed to air-dry. The stained smears were examined microscopically using a 100 × oil immersion objective (1,000 × total magnification). Each slide was systematically screened across the entire smear for the presence of trypanosomes. Bat fly specimens were subsequently examined under a stereomicroscope and identified to family level (e.g., Nycteribiidae, Streblidae) based on morphological characteristics using published taxonomic keys [26]. DNA extracted from selected ectoparasite specimens was subjected to PCR amplification of the mitochondrial *cox1* gene to confirm and, where possible, refine these identifications.

### Molecular methods

Genomic DNA was extracted from EDTA-preserved blood using the QIAamp Mini Kit (Qiagen) following the manufacturer’s protocol. DNA was eluted in 100 µL of elution buffer and stored at −20 °C. Genomic DNA from ethanol-preserved ectoparasites was extracted using the Quick-DNA Miniprep Plus Kit (Zymo Research). Ectoparasites were rinsed three times in sterile phosphate-buffered saline (PBS), air-dried under sterile conditions, and individually homogenized in 1.5 mL tubes using sterile pestles. Homogenates were incubated with 95 µL nuclease-free water, 95 µL solid tissue buffer and 10 µL Proteinase K at 55 °C for 5 h before further processing according to the manufacturer’s protocol. DNA was eluted in 100 µL elution buffer and stored at −20 °C.

PCR amplification was performed using the AllTaq Master Mix Kit (Qiagen) with 4–5 µL of genomic DNA and 1 µL of each primer (10 µM). Trypanosome screening employed a nested PCR targeting approximately 600 bp of the small subunit 18S ribosomal RNA gene (18S rRNA) following [27]. The primary reaction used primers TRY927F/R, and the nested reaction used SSU561F/R.

Selected ectoparasites were genotyped using the standard DNA barcoding primers LCO1490/HCO2198 [28], amplifying approximately 720 bp of the mitochondrial *cox1* gene. Primer sequences are provided in S2 Table. PCR products of the expected size were visualized by agarose gel electrophoresis and purified using the QIAquick PCR Purification Kit (QIAGEN) according to the manufacturer’s instructions. Purified amplicons were subsequently subjected to Sanger sequencing in both directions using the same primers as for amplification. Sequencing was performed at LGC Genomics (Berlin, Germany).

### Nucleotide sequence analysis

Nucleotide sequences were manually curated in Geneious Prime 2024.04. Ambiguous base calls were coded using standard ambiguity codes. Samples with low-quality reads were re-amplified and sequenced where possible. Sequences exhibiting ambiguous signals, including double peaks, elevated background noise, or a high proportion of unresolved nucleotides, were considered low quality. Although some of these sequences showed highest similarity to trypanosome sequences in BLAST searches, they were omitted from the phylogenetic inference due to insufficient quality for reliable analyses. These samples were, however, retained as positive detections for the purpose of prevalence estimation. Sequence identity was assessed using BLASTn (NCBI).

Mitochondrial *cox1* gene sequences obtained from ectoparasites were used for molecular identification. Edited sequences were compared against the NCBI database using BLASTn to determine their closest matches and confirm taxonomic assignment. Sequence identity values and top hits were used to support species-level or genus-level identification.

Trypanosome sequences were aligned using MAFFT implemented in *Geneious Prime* [29,30]. Reference sequences were retrieved from GenBank and included in the 18S rRNA alignment (accession numbers are provided in the respective phylogenetic tree figure and S3 Table). The final 18S rRNA dataset comprised 98 sequences (including 16 representative sequences from this study) with a total length of 821 nucleotides (nt) including 139 gap positions. The final alignment used for phylogenetic analysis is available in FASTA format from the OSF repository. Model selection was performed using *ModelTest-NG* 0.1.7 [31] implemented in *raxmlGUI v2.0.14* [32]. Maximum Likelihood (ML) analysis was conducted under the model TIM3 + I (proportion of invariant) + Gamma (rate heterogeneity). *Trypanosoma microti* and *Trypanosoma lewisi* were used as outgroups. Node support was assessed with 1,000 bootstrap replicates (thorough bootstrap). The phylogenetic tree was visualized in *FigTree* v.1.4.4 (http://tree.bio.ed.ac.uk/software/figtree/).

Putative molecular lineages were inferred using the Bayesian implementation of the Poisson Tree Processes model on the bPTP web server (https://species.h-its.org/ptp/). Because PTP-based approaches may overestimate the number of delimited lineages when terminal sampling is uneven [33], a reduced 18S rRNA dataset was generated for the bPTP analysis. The dataset was screened for identical and highly similar sequences, and phylogenetically redundant reference terminals from overrepresented species or lineages were pruned. Representative sequences from the major named species/lineages, the *T. livingstonei*/ *T.* cf*. livingstonei*-related clade, and all newly generated Nigerian sequences included in the analysis were retained. The final reduced alignment contained n = 65 terminals and had a length of 821 nt. A maximum-likelihood tree was re-estimated from this reduced alignment using the same settings as described above. The tree included selected outgroup taxa and was rooted using *Trypanosoma microti* and *Trypanosoma lewisi* as outgroups. The rooted tree was submitted to the bPTP server in Newick format, and the outgroup taxa were excluded from the delimitation analysis using the outgroup-removal option of the server. The bPTP analysis was run for 200,000 MCMC generations, with a thinning value of 100 and a burn-in of 25%. Convergence of the MCMC chain was assessed visually using the diagnostic plots provided by the server. The resulting groups were interpreted as putative molecular lineages/species-boundary hypotheses rather than formally recognized taxonomic species. The annotated bPTP output tree was visualized using the Interactive Tree of Life (iTOL) web server. Delimited lineages and bPTP support values/posterior probabilities were inspected on the annotated bPTP tree, both of which are among the PTP output files summarized in S2 File.

### Inclusivity in global research

Additional information regarding the ethical, cultural, and scientific considerations specific to inclusivity in global research is included in the Supporting Information (S1 File).

## Results

### Bat sampling and trypanosome prevalence

A total of 57 bats, representing ten species across five bat families (Hipposideridae, Molossidae, Nycteridae, Rhinolophidae, and Pteropodidae), were included in the analysis of trypanosome blood parasite infections. These individuals were captured from nine caves and a residential building. Species richness varied among roost sites, with Cave 5 and Cave 7 showing the highest number of recorded species. *Hipposideros abae* was the most frequently captured species (n = 23), followed by *Hipposideros ruber* (n = 11) and *Rhinolophus landeri* (n = 10). Several species were represented by single individuals (*Hipposideros jonesi, Nycteris arge, Nycteris grandis, Rhinolophus alcyone, Mops* sp.). Mixed-species aggregations involving *Hipposideros* spp. and *Rhinolophus* spp. were observed in several cave compartments, whereas *Nycteris* species were recorded in smaller or more spatially segregated groups. *Mops condylurus* was exclusively captured in the residential building.

Trypanosome infections were frequently detected using PCR screening, with an overall prevalence of 38.6% (22/57). A subset of sequences obtained from PCR-positive samples was excluded from phylogenetic analysis due to low sequence quality; however, these samples were retained as positive detections for prevalence estimation, as BLAST searches indicated highest similarity to trypanosome sequences. Infections occurred in six of the ten examined bat species (60% of species sampled): *Hipposideros abae* (11/23), *Hipposideros jonesi* (1/1), *Hipposideros ruber* (5/11), *Mops condylurus* (2/6), *Rhinolophus landeri* (2/10), and *Rousettus aegyptiacus* (1/2). The highest number of infected individuals was recorded for *H. abae*, which also represented the most frequently sampled species. In contrast, no infections were detected in *Nycteris arge* (0/1), *Nycteris grandis* (0/1), *Rhinolophus alcyone* (0/1), or *Mops* sp. (0/1) (Table 1); however, the very small sample sizes for these species limit the ability to draw conclusions about their general infection status.

**Table 1:** Overview of prevalence of trypanosome infections and ectoparasites from bats in Enugu State, Southeast Nigeria.

Bat species	Trypanosome infection prevalence, positive/examined (%)	Ectoparasite species	Frequency of ectoparasites (%)/Cave
*Rousettus aegyptiacus*	1/2 (50.0)	*Cyclopodia greeffi*	1 (12.5)/Cave 3
*Hipposideros abae*	11/23 (47.8)	*Cyclopodia greeffi*	1 (12.5)/Cave 6
		*Eucampsipoda africana*	2 (25.0)/Caves 5,8
*Hipposideros jonesi*	1/1 (100.0)	*0*	0
*Hipposideros ruber*	5/11 (45.5)	*Cyclopodia greeffi*	1 (12.5)/Cave 7
		*Eucampsipoda africana*	1 (12.5)/Cave 7
*Rhinolophus landeri*	2/10 (20.0)	Streblidae sp.	1 (12.5)/Cave 5
*Rhinolophus alcyone*	0/1	Streblidae sp.	1 (12.5)/Cave 5
*Nycteris arge*	0/1	0	0
*Nycteris grandis*	0/1	0	0
*Mops* sp.	0/1	0	0
*Mops condylurus*	2/6 (33.3)	0	0
**Total**	**22/**57 **(38.6)**		**8 (14.0)**

Infections were detected across multiple bat families, indicating that trypanosomes were not restricted to a single phylogenetic lineage within the sampled community. No trypanosomes were observed in blood smears by microscopy despite thorough microscopic screening, which may be consistent with low parasitemia in infected individuals.

### Spatial distribution of infections

**Fig 2:**
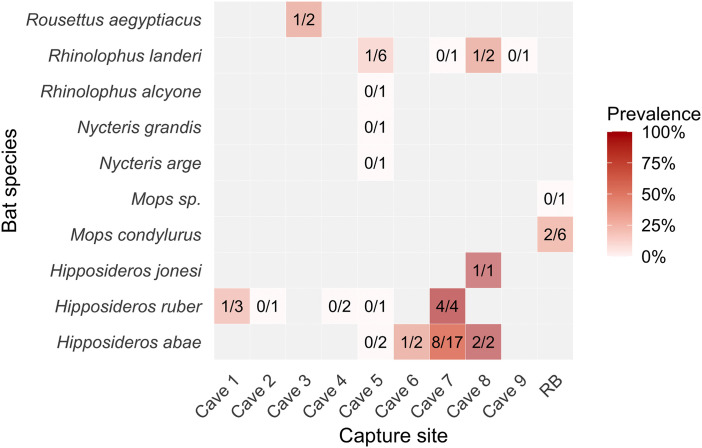
Site-specific prevalence of trypanosome infections by bat species and cave. Prevalence values represent the proportion of infected individuals per species within each sampling site and may differ from overall species-level prevalence values reported across all sites in the main text. The figure was generated in R version 4.3.1 using the packages *ggplot2, dplyr*, and *tidyr*. The full R script is available from the Open Science Framework (OSF) repository (DOI: 10.17605/OSF.IO/3794C).

Trypanosome infections were unevenly distributed among caves and bat species (Fig 2). Values shown in Fig 2 represent site-specific prevalence for each bat species, whereas prevalence values reported above reflect overall prevalence across all sampling sites. The highest diversity of infected host species was observed in Cave 7 and Cave 8. In Cave 7, infections were detected in *H. abae* (47.1%, 8/17) and *H. ruber* (100%, 4/4), representing both the highest number of infected individuals and the highest species co-occurrence of infected hosts within a single cave. Cave 8 harbored infected *H. abae* (100%, 2/2), *H. jonesi* (100%, 1/1), and *R. landeri* (50%, 1/2). In contrast, only a single infected species was recorded in Cave 3 (*R. aegyptiacus*, 50%, 1/2) and Cave 6 (*H. abae*, 50%, 1/2). Cave 5 exhibited the lowest overall prevalence (8.3%, 1/12), despite hosting multiple bat species. No trypanosome infections were detected in bats captured from Caves 2, 4, and 9. Infections were restricted to four of ten sampled roost sites, whereas no infections were detected in the remaining six sites. Thus, both infection prevalence and host diversity of infections varied markedly among roost sites. Due to uneven and small sample sizes across caves, no statistical comparison of prevalence among roost sites was performed.

### Molecular characterization of trypanosomes of bats in Southeastern Nigeria

Trypanosome DNA was detected by PCR and characterized by sequencing of the 18S rRNA gene. Sequences were compared to reference sequences in GenBank using the BLASTn algorithm. Several samples yielded lower-quality sequences that showed highest similarity to trypanosomes but could not be confidently assigned to a defined species. These sequences were excluded from subsequent phylogenetic analyses (S3 Table).

**Fig 3:**
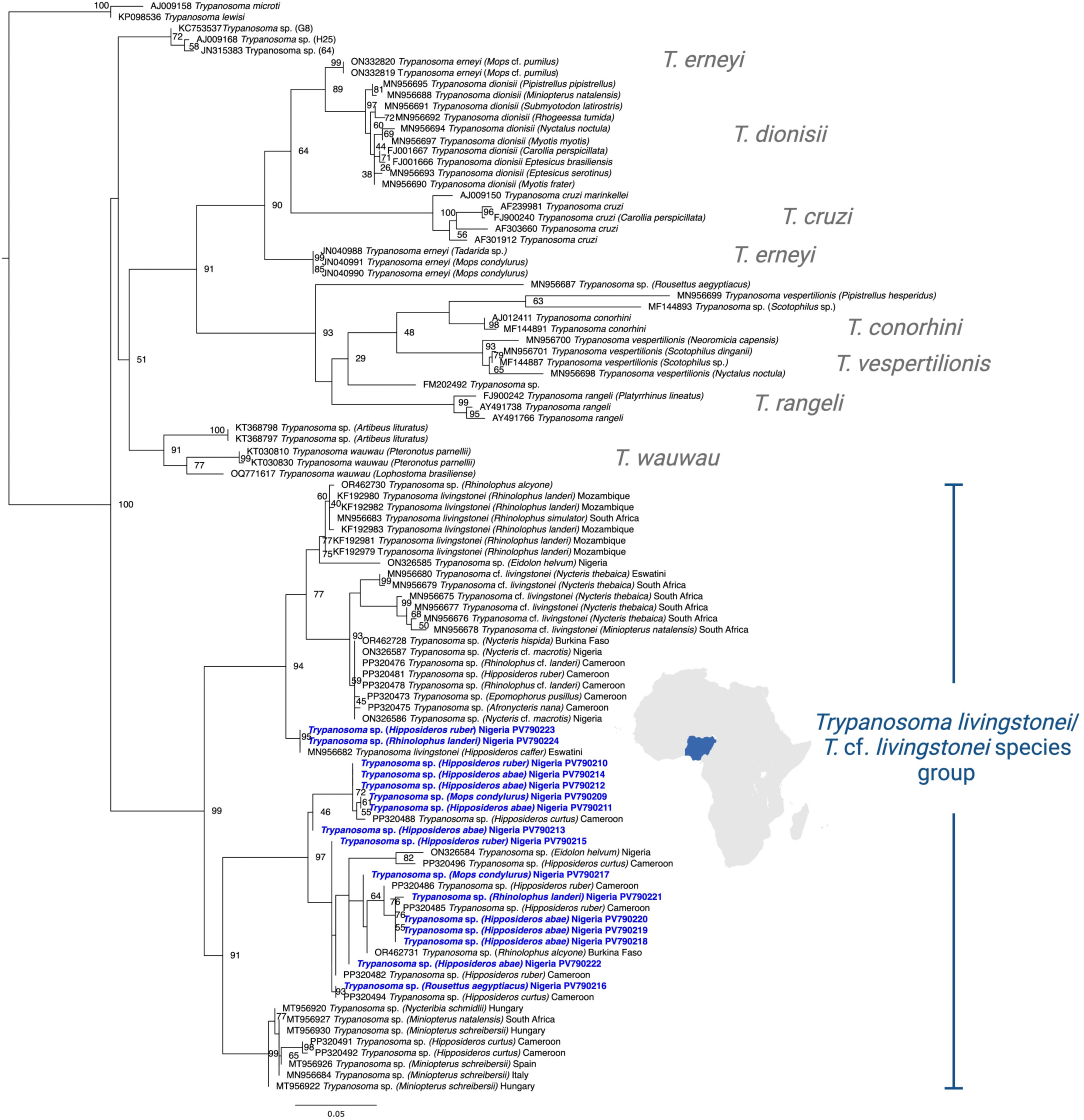
Maximum likelihood phylogeny of trypanosomes. The analysis is based on the 18S rRNA alignment that features a total length of 821 bp. *Trypanosoma lewisi* and *Trypanosoma microti* were used as outgroups. ML bootstrap values > 40 (using 1,000 replicates) are displayed at the nodes. Trypanosome sequences generated in this study are highlighted in blue and group within the broader *T. livingstonei/ T.* cf. *livingstonei*-related clade.

High-quality 18S rRNA trypanosome sequences from 16 bat samples were included in the phylogenetic analyses. All showed highest similarity to members of the *Trypanosoma livingstonei* species group [6,8,10,34]. Maximum likelihood phylogenetic analysis confirmed this initial identification and placed all sequences generated in this study within the broader *T. livingstonei* species group with strong support (bootstrap value of 99) (Fig 3). This clade predominantly comprises trypanosomes detected in diverse African bat species.

Within the *T. livingstonei* species group, two major subclades were resolved. One subclade includes reference sequences assigned to *T. livingstonei*, *T.* cf. *livingstonei,* and related *Trypanosoma* sp. sequences from Burkina Faso, Nigeria, and Cameroon. Two sequences from this study, one from *H. ruber* and one from *R. landeri*, grouped basal to this subclade together with a *T. livingstonei* sequence from *Hipposideros caffer* from Eswatini, with strong support (bootstrap = 94), and thus did not cluster with the remaining Nigerian sequences. The second major subclade comprises two further sequence clusters. The remaining Nigerian sequences from this study clustered within one of these groups, together with bat-associated *Trypanosoma* reference sequences. The other group included two sequences from *H. curtus* from Cameroon and sequences from European bat-associated trypanosomes.

The reduced bPTP analysis recovered 27 putative molecular lineages in the most supported partition (S1 Fig; S2 File). Of these, 12 putative molecular lineages were located within the broader *T. livingstonei*/ *T.* cf*. livingstonei*-related clade. Within this part of the tree, the analysis did not recover all sequences as a single homogeneous lineage. Instead, *T. livingstonei*- and *T.* cf*. livingstonei*-related reference sequences, together with the newly generated Nigerian sequences assigned to this clade, were distributed across several bPTP-delimited putative molecular lineages. Because several bPTP-delimited lineages received low to moderate support, the resulting groups are interpreted only as putative molecular lineages rather than as formally recognized taxonomic species. Overall, the molecular data indicate that bat-associated trypanosomes detected in southeastern Nigeria belong to the *T. livingstonei* species group but exhibit phylogenetic structuring within this clade. Despite extensive screening of blood smears, no trypanosome parasite stages were detected, precluding morphological characterization of the molecularly detected parasites.

### Ectoparasite occurrence and genotyping

Three dipteran ectoparasite taxa were identified across the sampled bats. Nycteribiid bat flies (*Cyclopodia greeffi*, n = 3 and *Eucampsipoda africana,* n = 3) and two streblid flies (Streblidae sp.) were detected on multiple host species, alongside a single mite collected from *Hipposideros abae* (Table 1). *H. abae* hosted the highest diversity of ectoparasites, including both nycteribiid species and the mite, whereas streblid flies were observed on *Rhinolophus landeri* and *Rhinolophus alcyone.*

Molecular identification confirmed three nycteribiid flies as *Cyclopodia greeffi*, collected from *H. abae* (n = 1), *H. ruber* (n = 1), and *Rousettus aegyptiacus* (n = 1), showing 99.8–100% nucleotide identity to reference sequence ON324536 (collected from *Eidolon helvum* in Nigeria). Three additional nycteribiid flies were identified as *Eucampsipoda africana*, collected from *H. abae* (n = 2; 100% identity to MH151064 from *R. aegyptiacus* in Nigeria) and *H. ruber* (n = 1; 99.8% identity to KR997992 from *R. aegyptiacus* in South Africa).

Two streblid flies, one from *R. landeri* and one from *R. alcyone*, showed highest similarity (89% nucleotide identity) to a Streblidae sp. sequence (MW792206) from *H. ruber* in Uganda. The mite collected from *H. abae* showed 85% nucleotide identity to members of the genus *Spinturnix*, with highest similarity to a mite sequence obtained from a *Myotis* bat in Colombia (PP967723).

Nycteribiid ectoparasites were additionally screened for trypanosome DNA; however, no infections were detected. GenBank accession numbers for all arthropod sequences generated in this study are provided in Supplementary S3 Table.

## Discussion

In this study, 38.6% of bats captured from caves located near human habitations and a residential building in Enugu State, southeastern Nigeria, tested positive for DNA of trypanosomes assigned to the *T. livingstonei* species group. This represents the first genetic characterization of trypanosome infections in cave-dwelling bats from southern Nigeria and provides new insights into the prevalence and phylogenetic diversity of these bat-associated trypanosomes in this understudied region.

Trypanosome infections were detected in six bat species across four families, indicating that these parasites are broadly distributed within the local bat community. The relatively high prevalence observed in hipposiderid bats, particularly *H. abae* and *H. ruber*, is consistent with previous studies identifying trypanosomes in hipposiderid hosts across Africa [7,8,10]. The detection of infections in multiple bat families further supports the capacity of bat-associated trypanosomes to infect phylogenetically diverse hosts.

Species composition and apparent infection patterns differed among roost sites; however, no formal statistical analysis was performed to compare prevalence among caves due to small and uneven sample sizes across caves and host species, and these observations should therefore be interpreted with caution. Infections were detected in multiple caves, including Caves 7 and 8, but the extent to which this reflects true spatial variation versus host composition or sampling effort remains unclear (Fig 2). Caves 7 and 8 (Ogba-ububa and Ogba-nekwuokwu) appeared to differ from the other sampled caves in several qualitative ecological aspects, including larger size, greater structural complexity, and higher apparent levels of anthropogenic disturbance. However, the role of such site-level characteristics in shaping parasite occurrence cannot be determined from the present data. Although a comparatively higher number of infected individuals was observed in these caves, this pattern likely reflects the distribution and abundance of host species rather than cave-specific environmental conditions. For example, infections were primarily detected in a subset of bat species across multiple caves, while sites with high species richness such as Cave 5, did not necessarily exhibit higher infection levels. Overall, the observed variation in trypanosome detection is more consistent with host species identity and uneven sampling effort than with ecological differences among caves. Consequently, the present study primarily documents the host range of trypanosomes within the sampled bat community, and further work incorporating standardized seasonal sampling and quantitative ecological measurements would be required to test for environmental drivers of infection.

The ecological heterogeneity of southeastern Nigeria likely contributes to the observed bat community composition across roost sites. Previous studies have demonstrated that bat diversity in West Africa is closely linked to the region’s complex habitat mosaics and landscape structure [35]. The variation in species richness among caves in the present study is consistent with this broader regional pattern. *Hipposideros* spp. and *Rhinolophus* spp. commonly roost in dense aggregations and were frequently observed in shared cave compartments. Elevated contact rates in these mixed-species aggregations may increase opportunities for parasite transmission and could contribute to the comparatively higher prevalence observed in hipposiderid taxa [36,37]. In contrast, *Nycteris* spp., which were not infected in this study, were observed in smaller or more spatially segregated groups. Although *Nycteris* spp. can form mixed-species roosts, field observations in the sandstone cave systems indicated a tendency toward smaller, single-species aggregations for members of this family. Such roosting behavior may limit direct contact opportunities and influence transmission dynamics. However, the very small sample sizes for these taxa (often n = 1) limit the ability to draw conclusions about their general infection status. Similarly, *Mops condylurus*, which roosts in residential buildings and can form large colonies [23], showed lower infection levels in this study, indicating that host ecology alone may not fully explain prevalence differences. Together, these patterns highlight the importance of integrating roost structure, colony size, and host identity when assessing parasite dynamics in cave systems.

All trypanosome sequences generated in this study clustered within the *T. livingstonei* species group, supporting the broad distribution of this group among African bats. Phylogenetic analyses revealed structured subclades within the broader *T. livingstonei*/ *T.* cf. *livingstonei*-related clade, consistent with previous studies reporting considerable genetic diversity within this group [8]. Most Nigerian sequences grouped within one part of this clade together with bat-associated *Trypanosoma* reference sequences, whereas two Nigerian sequences formed a more basal grouping together with a *T. livingstonei* from southern Africa. This pattern suggests that the Nigerian bat trypanosomes are not phylogenetically homogeneous and may reflect broader geographic or host-associated structuring within the *T. livingstonei* species group.

Previous species-delimitation analyses of the *T. cruzi* clade identified *T.* cf*. livingstonei* as a divergent lineage closely related to *T. livingstonei*, but its species-level status remained method-dependent [8]. Clément et al. [8] reported that GMYC separated *T.* cf*. livingstonei* from *T. livingstonei*, whereas mPTP and ABGD grouped them as a single taxon, and therefore treated *T.* cf*. livingstonei* conservatively as a subspecies rather than as a distinct species. In our reduced bPTP analysis, the broader *T. livingstonei*/ *T. cf. livingstonei*-related clade was also not recovered as a single homogeneous lineage. Instead, 12 of the 27 putative molecular lineages recovered in the most supported partition were located within this part of the tree, including several lineages containing newly generated Nigerian sequences.

These results suggest additional molecular lineage structure within the *T. livingstonei*/ *T.* cf*. livingstonei*-related clade after inclusion of the Nigerian sequences. However, several of the relevant bPTP-delimited lineages received only low to moderate support. Thus, the bPTP-delimited groups are interpreted as putative molecular lineages that require further investigation.

A limitation of the present study is that phylogenetic and PTP analyses were based on a partial 18S rRNA gene fragment (~600 bp), which provides limited resolution for distinguishing closely related *Trypanosoma* lineages, particularly within the *T. cruzi* clade. Attempts to amplify longer 18S rRNA fragments and additional loci were unsuccessful due to limited DNA quantity and quality, further constraining the achievable resolution. Future studies incorporating longer SSU rRNA sequences and additional genetic markers, such as gGAPDH or kinetoplast-encoded genes, together with broader geographic and host sampling, will be necessary to test whether the bPTP-delimited lineages correspond to independently evolving species.

The occurrence of *T. livingstonei* across multiple bat families, including Molossidae in this study, underscores the broad host range of this parasite group. Given that African bat trypanosomes are considered basal within the *T. cruzi* clade [7,8,11], continued molecular characterization of bat-associated trypanosomes is essential for clarifying their evolutionary history and diversification. Although no trypanosome stages were detected in blood smears, molecular analyses clearly demonstrate the circulation of genetically diverse lineages within cave-roosting bat populations in southeastern Nigeria.

Ectoparasite screening revealed the presence of nycteribiid and streblid flies as well as a mite associated with sampled bats. Molecular identification confirmed high similarity of *Cyclopodia greeffi* and *Eucampsipoda africana* to previously reported Nigerian sequences (ON324536, MH151064), supporting their wide ecological distribution and host associations [38,39]. The comparatively low ectoparasite diversity observed may reflect ecological characteristics of the caves, seasonal effects, or sampling limitations.

None of the screened ectoparasites tested positive for trypanosome DNA. While this does not exclude their potential role in parasite transmission, it suggests that further targeted sampling with larger ectoparasite sample sizes is required to clarify their involvement. The transmission routes of most African bat-associated trypanosomes remain poorly understood, with several potential pathways suggested but not yet clearly resolved. For example, *Trypanosoma leleupi* has been shown to be transmitted by cimicid bat bugs (*Stricticimex brevispinosus* and *Afrocimex leleupi*) [40]. Cimicid bat bugs have also been shown to transmit trypanosomes in European *Pipistrellus* bats [41,42]. More broadly, cimicid bat bugs are considered vectors of several bat-associated trypanosomes, including *T. dionisii* and *T. (Megatrypanum) incertum*, in Africa and Europe, and the bat-restricted species *Stricticimex brevispinosus* has been found infected with *Megatrypanum* trypanosomes in Africa [43,44]. In contrast, the vectors of *T. livingstonei*, the lineage detected in this study, remain unknown, and experimental evidence suggests that triatomine bugs are unlikely to serve as vectors, consistent with their absence in Africa [7]. In addition, trypanosome DNA has been detected in bat-associated ectoparasites, including nycteribiid and streblid flies, indicating that these arthropods may reflect host infections or potentially contribute to transmission; however, their role as confirmed biological vectors remains unproven [45]. Beyond vector-borne transmission, alternative pathways may also contribute to parasite circulation in bat populations. Oral transmission, for instance through the ingestion of infected ectoparasites by insectivorous bats or via grooming behavior, has been proposed as an important mechanism facilitating transmission within the *T. cruzi* clade [7,43]. These observations indicate that bat-associated trypanosomes may be maintained through a combination of transmission routes, including both vectorial and non-vectorial pathways. Nevertheless, the relative importance of these mechanisms in African bat systems remains unclear. Future studies are needed that integrate sampling of bats and their associated ectoparasites, particularly cimicid bugs and bat flies, to better resolve transmission dynamics.

Cave systems in the study area exhibited considerable variation in bat species composition and capture rates, indicating that environmental and structural characteristics likely shape bat occupancy and community structure. Differences in cave morphology, microclimate, and habitat heterogeneity may influence both host assemblages and opportunities for parasite transmission. Similar patterns have been reported from other regions, where cave-dwelling bat assemblages harbor diverse trypanosomatid lineages, suggesting that cave environments may facilitate parasite maintenance and transmission through high host density and frequent interspecific contact [46].

Thus, both cave-level ecological parameters and host species identity likely contribute to the observed heterogeneity in infection prevalence. Future studies incorporating detailed ecological assessments of cave environments and longitudinal sampling would help disentangle site-specific from host-specific drivers of infection.

Overall, this study expands current knowledge of cave-roosting bat–trypanosome associations in southeastern Nigeria. By linking infection prevalence, host diversity, and phylogenetic placement within the *T. livingstonei* complex, the findings highlight the ecological complexity of bat–parasite interactions in cave systems and underscore the importance of integrating host community structure and roost ecology into studies of parasite diversity and evolution.

## Conclusions

Bats roosting in peri-domestic caves and residential structures in southeastern Nigeria harbor genetically diverse members of the *Trypanosoma livingstonei* species group. This study provides the first molecular characterization of trypanosome infections in cave-dwelling bats from southern Nigeria and documents their occurrence across multiple bat families within heterogeneous roost systems. The observed variation in species composition and infection prevalence among caves highlights the importance of integrating host community structure and roost ecology when investigating parasite dynamics in bat populations. Expanded geographic sampling, multilocus phylogenetic analyses, and ecological characterization of cave habitats will be essential to further resolve lineage diversity and clarify the ecological drivers shaping bat–trypanosome associations in understudied regions of West Africa.

## Supporting information

S1 Fig Annotated bPTP species-delimitation tree based on the 18S rRNA maximum-likelihood phylogeny. The tree was visualized in iTOL. The reduced bPTP analysis recovered 27 putative molecular lineages in the most supported partition. Newly generated Nigerian sequences are highlighted in blue, and colored strips indicate their assignment, together with related reference sequences, to bPTP-delimited lineages within the *Trypanosoma livingstonei*/ *T.* cf*. livingstonei*-related clade. Values shown within the tree indicate branch support values and are displayed only when ≥ 0.4 (full list of lineages and support values are listed in S2 File). Values associated with the colored strips indicate bPTP support values/posterior probabilities for the corresponding delimited lineages. Because several bPTP-delimited lineages received low to moderate support, the highlighted groups are interpreted as exploratory species-boundary hypotheses rather than formally recognized species.
(PDF)

S1 Table Overview of investigated bats and infections with trypanosomes. (DOCX)

S2 Table Nucleotide primers used in this study. (DOCX)

S3 Table GenBank accession numbers for *Trypanosoma* parasites (18S rRNA) and arthropods of the study. (DOCX)

S1 File PLOS Inclusivity in Global Research questionnaire. The completed PLOS Inclusivity in Global Research questionnaire is provided as S1 File.
(DOCX)

S2 File Raw bPTP output tree. The file contains the annotated output tree generated by the bPTP web server for the reduced 18S rRNA dataset used in the final species-delimitation analysis. The most supported partition recovered 27 putative molecular lineages. The corresponding iTOL visualization focusing on the *Trypanosoma livingstonei*/ *T*. cf. *livingstonei*-related clade is shown in S1 Fig.
(PDF)
