# Supplementary material for: Cave-dwelling bats as hosts of Trypanosoma livingstonei: Evidence from peri-domestic roosts in southeastern Nigeria
Source: PLoS One. 2026 Aug 12;21(8):e0355840. doi: 10.1371/journal.pone.0355840 (PMC13465849; doi:10.1371/journal.pone.0355840)
Supplement: Supplementary file 1 [file pone.0355840.s001.pdf]

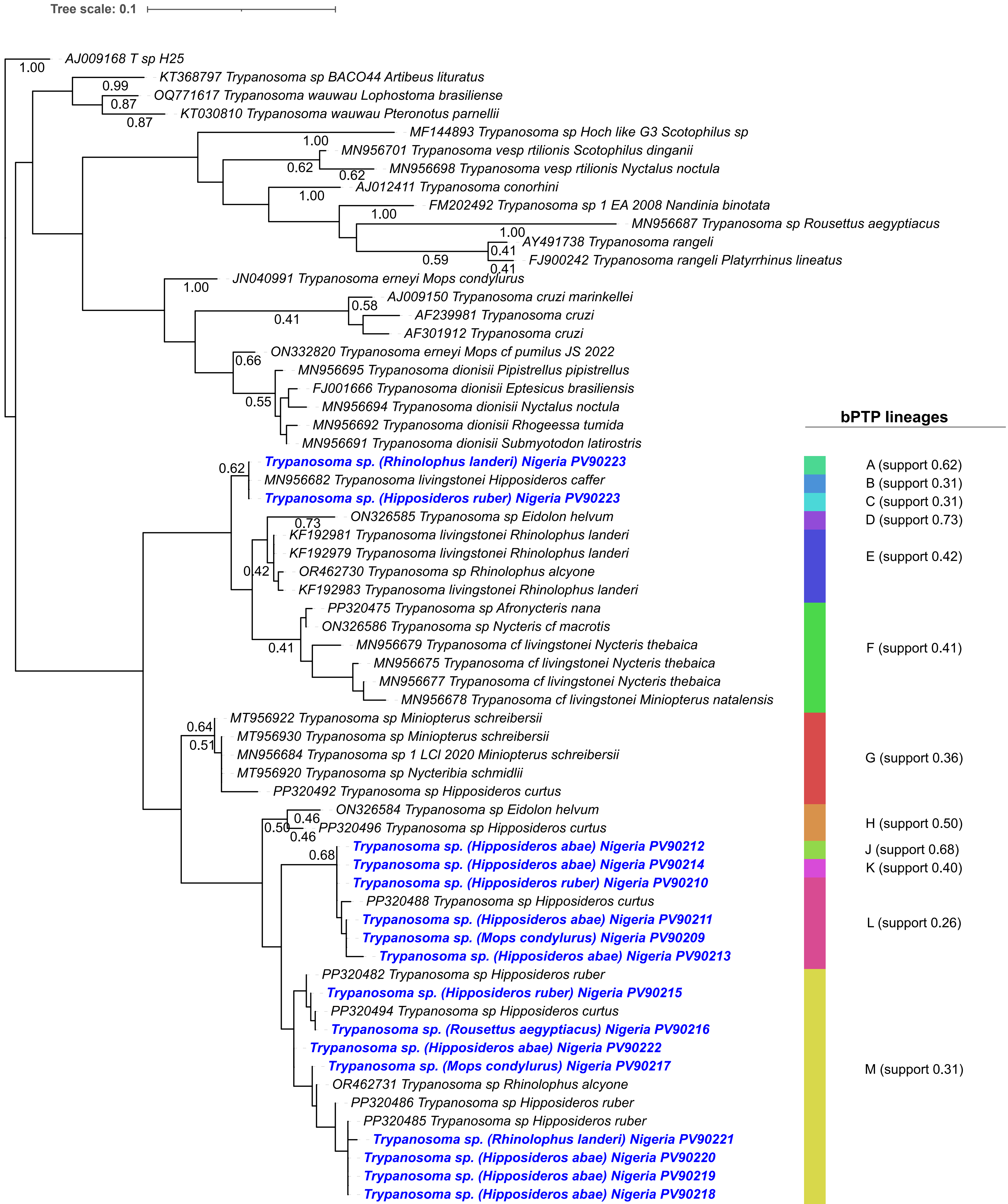

Tree scale: 0.1
AJ009168 T sp H25
KT368797 Trypanosoma sp BACO44 Artibeus lituratus
OQ771617 Trypanosoma wauwau Lophostoma brasiliense
KT030810 Trypanosoma wauwau Pteronotus parnellii
MF144893 Trypanosoma sp Hoch like G3 Scotophilus sp
MN956701 Trypanosoma vesp rtilionis Scotophilus dinganii
MN956698 Trypanosoma vesp rtilionis Nyctalus noctula
AJ012411 Trypanosoma conorhini
FM202492 Trypanosoma sp 1 EA 2008 Nandinia binotata
MN956687 Trypanosoma sp Rousettus aegyptiacus
AY491738 Trypanosoma rangeli
FJ900242 Trypanosoma rangeli Platyrrhinus lineatus
JN040991 Trypanosoma erneyi Mops condylurus
AJ009150 Trypanosoma cruzi marinkellei
AF239981 Trypanosoma cruzi
AF301912 Trypanosoma cruzi
ON332820 Trypanosoma erneyi Mops cf pumilus JS 2022
MN956695 Trypanosoma dionisii Pipistrellus pipistrellus
FJ001666 Trypanosoma dionisii Eptesicus brasiliensis
MN956694 Trypanosoma dionisii Nyctalus noctula
MN956692 Trypanosoma dionisii Rhogeessa tumida
MN956691 Trypanosoma dionisii Submyotodon latirostris
Trypanosoma sp. (Rhinolophus landeri) Nigeria PV90223
MN956682 Trypanosoma livingstonei Hipposideros caffer
Trypanosoma sp. (Hipposideros ruber) Nigeria PV90223
ON326585 Trypanosoma sp Eidolon helvum
KF192981 Trypanosoma livingstonei Rhinolophus landeri
KF192979 Trypanosoma livingstonei Rhinolophus landeri
OR462730 Trypanosoma sp Rhinolophus alcyone
KF192983 Trypanosoma livingstonei Rhinolophus landeri
PP320475 Trypanosoma sp Afronycteris nana
ON326586 Trypanosoma sp Nycteris cf macrotis
MN956679 Trypanosoma cf livingstonei Nycteris thebaica
MN956675 Trypanosoma cf livingstonei Nycteris thebaica
MN956677 Trypanosoma cf livingstonei Nycteris thebaica
MN956678 Trypanosoma cf livingstonei Miniopterus natalensis
MT956922 Trypanosoma sp Miniopterus schreibersii
MT956930 Trypanosoma sp Miniopterus schreibersii
MN956684 Trypanosoma sp 1 LCI 2020 Miniopterus schreibersii
MT956920 Trypanosoma sp Nycteribia schmidlii
PP320492 Trypanosoma sp Hipposideros curtus
ON326584 Trypanosoma sp Eidolon helvum
PP320496 Trypanosoma sp Hipposideros curtus
Trypanosoma sp. (Hipposideros abae) Nigeria PV90212
Trypanosoma sp. (Hipposideros abae) Nigeria PV90214
Trypanosoma sp. (Hipposideros ruber) Nigeria PV90210
PP320488 Trypanosoma sp Hipposideros curtus
Trypanosoma sp. (Hipposideros abae) Nigeria PV90211
Trypanosoma sp. (Mops condylurus) Nigeria PV90209
Trypanosoma sp. (Hipposideros abae) Nigeria PV90213
PP320482 Trypanosoma sp Hipposideros ruber
Trypanosoma sp. (Hipposideros ruber) Nigeria PV90215
PP320494 Trypanosoma sp Hipposideros curtus
Trypanosoma sp. (Rousettus aegyptiacus) Nigeria PV90216
Trypanosoma sp. (Hipposideros abae) Nigeria PV90222
Trypanosoma sp. (Mops condylurus) Nigeria PV90217
OR462731 Trypanosoma sp Rhinolophus alcyone
PP320486 Trypanosoma sp Hipposideros ruber
PP320485 Trypanosoma sp Hipposideros ruber
Trypanosoma sp. (Rhinolophus landeri) Nigeria PV90221
Trypanosoma sp. (Hipposideros abae) Nigeria PV90220
Trypanosoma sp. (Hipposideros abae) Nigeria PV90219
Trypanosoma sp. (Hipposideros abae) Nigeria PV90218
bPTP lineages
A (support 0.62)
B (support 0.31)
C (support 0.31)
D (support 0.73)
E (support 0.42)
F (support 0.41)
G (support 0.36)
H (support 0.50)
J (support 0.68)
K (support 0.40)
L (support 0.26)
M (support 0.31)
