# Supplementary material for: Cave-dwelling bats as hosts of Trypanosoma livingstonei: Evidence from peri-domestic roosts in southeastern Nigeria
Source: PLoS One. 2026 Aug 12;21(8):e0355840. doi: 10.1371/journal.pone.0355840 (PMC13465849; doi:10.1371/journal.pone.0355840)
Supplement: Supplementary file 2 [file pone.0355840.s006.pdf]

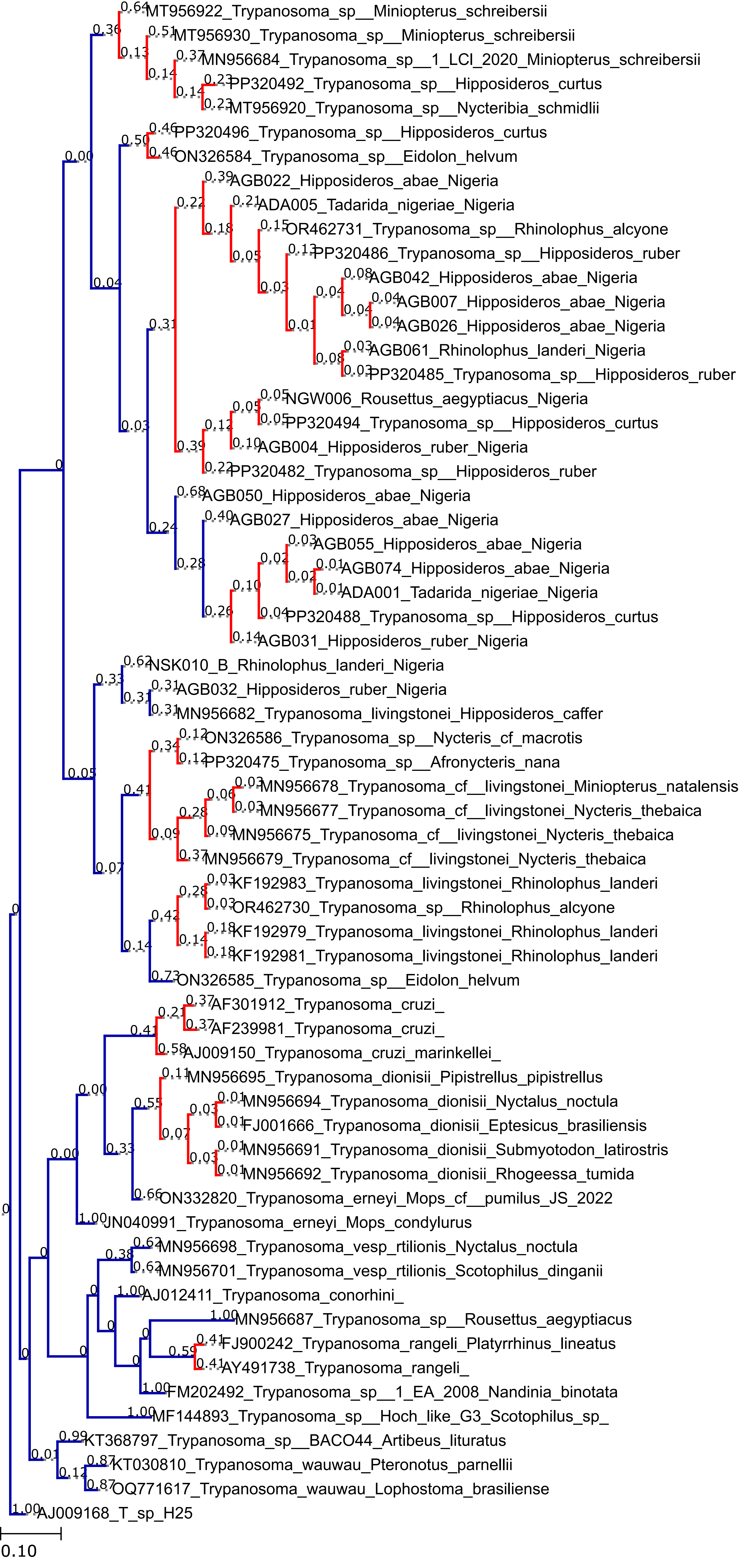
MT956922_Trypanosoma_sp__Miniopterus_schreibersii
MT956930_Trypanosoma_sp__Miniopterus_schreibersii
MN956684_Trypanosoma_sp__1_LCI_2020_Miniopterus_schreibersii
PP320492_Trypanosoma_sp__Hipposideros_curtus
MT956920_Trypanosoma_sp__Nycteribia_schmidlii
PP320496_Trypanosoma_sp__Hipposideros_curtus
ON326584_Trypanosoma_sp__Eidolon_helvum
AGB022_Hipposideros_abae_Nigeria
ADA005_Tadarida_nigeriae_Nigeria
OR462731_Trypanosoma_sp__Rhinolophus_alcyone
PP320486_Trypanosoma_sp__Hipposideros_ruber
AGB042_Hipposideros_abae_Nigeria
AGB007_Hipposideros_abae_Nigeria
AGB026_Hipposideros_abae_Nigeria
AGB061_Rhinolophus_landeri_Nigeria
PP320485_Trypanosoma_sp__Hipposideros_ruber
NGW006_Rousettus_aegyptiacus_Nigeria
PP320494_Trypanosoma_sp__Hipposideros_curtus
AGB004_Hipposideros_ruber_Nigeria
PP320482_Trypanosoma_sp__Hipposideros_ruber
AGB050_Hipposideros_abae_Nigeria
AGB027_Hipposideros_abae_Nigeria
AGB055_Hipposideros_abae_Nigeria
AGB074_Hipposideros_abae_Nigeria
ADA001_Tadarida_nigeriae_Nigeria
PP320488_Trypanosoma_sp__Hipposideros_curtus
AGB031_Hipposideros_ruber_Nigeria
NSK010_B_Rhinolophus_landeri_Nigeria
AGB032_Hipposideros_ruber_Nigeria
MN956682_Trypanosoma_livingstonei_Hipposideros_caffer
ON326586_Trypanosoma_sp__Nycteris_cf_macrotis
PP320475_Trypanosoma_sp__Afronycteris_nana
MN956678_Trypanosoma_cf__livingstonei_Miniopterus_natalensis
MN956677_Trypanosoma_cf__livingstonei_Nycteris_thebaica
MN956675_Trypanosoma_cf__livingstonei_Nycteris_thebaica
MN956679_Trypanosoma_cf__livingstonei_Nycteris_thebaica
KF192983_Trypanosoma_livingstonei_Rhinolophus_landeri
OR462730_Trypanosoma_sp__Rhinolophus_alcyone
KF192979_Trypanosoma_livingstonei_Rhinolophus_landeri
KF192981_Trypanosoma_livingstonei_Rhinolophus_landeri
ON326585_Trypanosoma_sp__Eidolon_helvum
AF301912_Trypanosoma_cruzi_
AF239981_Trypanosoma_cruzi_
AJ009150_Trypanosoma_cruzi_marinkellei_
MN956695_Trypanosoma_dionisii_Pipistrellus_pipistrellus
MN956694_Trypanosoma_dionisii_Nyctalus_noctula
FJ001666_Trypanosoma_dionisii_Eptesicus_brasiliensis
MN956691_Trypanosoma_dionisii_Submyotodon_latirostris
MN956692_Trypanosoma_dionisii_Rhogeessa_tumida
ON332820_Trypanosoma_erneyi_Mops_cf__pumilus_JS_2022
JN040991_Trypanosoma_erneyi_Mops_condylurus
MN956698_Trypanosoma_vesp_rtilionis_Nyctalus_noctula
MN956701_Trypanosoma_vesp_rtilionis_Scotophilus_dinganii
AJ012411_Trypanosoma_conorhini_
MN956687_Trypanosoma_sp__Rousettus_aegyptiacus
FJ900242_Trypanosoma_rangeli_Platyrrhinus_lineatus
AY491738_Trypanosoma_rangeli_
FM202492_Trypanosoma_sp__1_EA_2008_Nandinia_binotata
MF144893_Trypanosoma_sp__Hoch_like_G3_Scotophilus_sp_
KT368797_Trypanosoma_sp__BACO44_Artibeus_lituratus
KT030810_Trypanosoma_wauwau_Pteronotus_parnellii
OQ771617_Trypanosoma_wauwau_Lophostoma_brasiliense
AJ009168_T_sp_H25
0.10
