# Supplementary material for: Cave-dwelling bats as hosts of Trypanosoma livingstonei: Evidence from peri-domestic roosts in southeastern Nigeria
Source: PLoS One. 2026 Aug 12;21(8):e0355840. doi: 10.1371/journal.pone.0355840 (PMC13465849; doi:10.1371/journal.pone.0355840)
Supplement: Supplementary file 3 [file pone.0355840.s002.docx]

**S1 Table. Overview of investigated bats and infections with trypanosomes.**

| **Sample No.** | **Bat species** | **Specimen deposition in SMACON museum/released** | **Feeding habit** | **Sex** | **Trypanosome infection** |
| --- | --- | --- | --- | --- | --- |
| AGB001 | *Hipposideros abae* | released | insectivorous | male | no |
| AGB007 | *Hipposideros abae* | released | insectivorous | female | yes |
| AGB009 | *Hipposideros abae* | released | insectivorous | female | no |
| AGB010 | *Hipposideros abae* | released | insectivorous | male | no |
| AGB011 | *Hipposideros abae* | released | insectivorous | male | yes |
| AGB016 | *Hipposideros abae* | released | insectivorous | male | yes |
| AGB017 | *Hipposideros abae* | released | insectivorous | male | no |
| AGB019 | *Hipposideros abae* | released | insectivorous | male | no |
| AGB022 | *Hipposideros abae* | released | insectivorous | male | yes |
| AGB026 | *Hipposideros abae* | released | insectivorous | female | yes |
| AGB027 | *Hipposideros abae* | released | insectivorous | male | yes |
| AGB028 | *Hipposideros abae* | released | insectivorous | male | no |
| AGB030 | *Hipposideros abae* | released | insectivorous | male | no |
| AGB034 | *Hipposideros abae* | released | insectivorous | female | no |
| AGB042 | *Hipposideros abae* | released | insectivorous | female | yes |
| AGB050 | *Hipposideros abae* | released | insectivorous | female | yes |
| AGB054 | *Hipposideros abae* | released | insectivorous | female | no |
| AGB055 | *Hipposideros abae* | released | insectivorous | male | yes |
| AGB074 | *Hipposideros abae* | released | insectivorous | female | yes |
| ENE003 | *Hipposideros abae* | released | insectivorous | male | yes |
| ENE004 | *Hipposideros abae* | released | insectivorous | female | no |
| NSK008 | *Hipposideros abae* | voucher | insectivorous | male | no |
| NSK021 | *Hipposideros abae* | voucher | insectivorous | male | no |
| AGB066 | *Hipposideros jonesi* | released | insectivorous | male | yes |
| AGB004 | *Hipposideros ruber* | released | insectivorous | female | yes |
| AGB008 | *Hipposideros ruber* | released | insectivorous | female | yes |
| AGB031 | *Hipposideros ruber* | released | insectivorous | male | yes |
| AGB032 | *Hipposideros ruber* | released | insectivorous | female | yes |
| AWH004 | *Hipposideros ruber* | voucher | insectivorous | female | no |
| AWH013 | *Hipposideros ruber* | released | insectivorous | female | no |
| AWH014 | *Hipposideros ruber* | released | insectivorous | female | no |
| NSK009 | *Hipposideros ruber* | released | insectivorous | male | no |
| OPI001 | *Hipposideros ruber* | released | insectivorous | male | no |
| OPI003 | *Hipposideros ruber* | released | insectivorous | male | no |
| NSK012 | *Rhinolophus landeri* | voucher | insectivorous | male | no |
| NSK018 | *Rhinolophus landeri* | voucher | insectivorous | male | no |
| ADA001 | *Mops condylurus* | released | insectivorous | male | yes |
| ADA002 | *Mops condylurus* | voucher | insectivorous | male | no |
| ADA005 | *Mops condylurus* | released | insectivorous | female | yes |
| ADA011 | *Mops condylurus* | released | insectivorous | male | no |
| ADA015 | *Mops sp.* | voucher | insectivorous | male | no |
| ADA019 | *Mops condylurus* | released | insectivorous | male | no |
| ADA020 | *Mops condylurus* | released | insectivorous | male | no |
| NSK014 | *Nycteris grandis* | voucher | insectivorous | female | no |
| NSK026 | *Nycteris arge* | released | insectivorous | male | no |
| NSK011 | *Rhinolophus alcyone* | released | insectivorous | male | no |
| AGB061 | *Rhinolophus landeri* | released | insectivorous | female | yes |
| AGB067 | *Rhinolophus landeri* | released | insectivorous | male | no |
| AWH011 | *Hipposideros ruber* | voucher | insectivorous | male | yes |
| NSK005 | *Rhinolophus landeri* | voucher | insectivorous | male | no |
| NSK010 | *Rhinolophus landeri* | voucher | insectivorous | male | yes |
| NSK017 | *Rhinolophus landeri* | voucher | insectivorous | male | no |
| NSK022 | *Rhinolophus landeri* | voucher | insectivorous | male | no |
| OGU004 | *Rhinolophus landeri* | voucher | insectivorous | female | no |
| AGB048 | *Rhinolophus landeri* | released | insectivorous | female | no |
| NGW005 | *Rousettus aegyptiacus* | released | frugivorous | male | no |
| NGW006 | *Rousettus aegyptiacus* | released | frugivorous | male | yes |
