# Supplementary material for: Cave-dwelling bats as hosts of Trypanosoma livingstonei: Evidence from peri-domestic roosts in southeastern Nigeria
Source: PLoS One. 2026 Aug 12;21(8):e0355840. doi: 10.1371/journal.pone.0355840 (PMC13465849; doi:10.1371/journal.pone.0355840)
Supplement: Supplementary file 4 [file pone.0355840.s003.docx]

**S2 Table. Nucleotide primers used in this study.**

| **Gene** | **Primer name** | **Sequence (5´- 3´)** | **Reference** |
| --- | --- | --- | --- |
| *18S rRNA*  (trypanosomes) | TRY927F | GAAACAAGAAACACGGGAG | Noyes et al. (1999) |
|  | TRY927R | CTACTGGGCAGCTTGGA | Noyes et al. (1999) |
|  | SSU561F | TGGGATAACAAAGGAGCA | Noyes et al. (1999) |
|  | SSU561R | CTGAGACTGTAACCTCAAAGC | Noyes et al. (1999) |
| ***cox1*** (arthropods) | LCO1490 | GGTCAACAAATCATAAAGATATTGG | Folmer et al. (1994) |
|  | HCO2198 | TAAACTTCAGGGTGACCAAAAAATCA | Folmer et al. (1994) |

*18S rRNA,* SSU rRNA, a component of the eukaryotic ribosomal *small* subunit
