# Supplementary material for: Cave-dwelling bats as hosts of Trypanosoma livingstonei: Evidence from peri-domestic roosts in southeastern Nigeria
Source: PLoS One. 2026 Aug 12;21(8):e0355840. doi: 10.1371/journal.pone.0355840 (PMC13465849; doi:10.1371/journal.pone.0355840)
Supplement: Supplementary file 5 [file pone.0355840.s004.docx]

**Table S3. GenBank accession numbers for *Trypanosoma* parasites (18S rRNA) and arthropods of the study.**

| **Sample number** | **Bat species** | ***Trypanosoma* species/arthropod species** | **GenBank accession number (18S rRNA)** |
| --- | --- | --- | --- |
| AGB007 | *Hipposideros abae* | *Trypanosoma* sp. A | PV790218 |
| AGB011 | *Hipposideros abae* | Low-quality sequence (closest BLAST match: *Trypanosoma* sp.) | - |
| AGB016 | *Hipposideros abae* | Low-quality sequence (closest BLAST match: *Trypanosoma* sp.) | - |
| AGB022 | *Hipposideros abae* | *Trypanosoma* sp. A | PV790222 |
| AGB026 | *Hipposideros abae* | *Trypanosoma* sp. A | PV790219 |
| AGB027 | *Hipposideros abae* | *Trypanosoma* sp. A | PV790214 |
| AGB042 | *Hipposideros abae* | *Trypanosoma* sp. A | PV790220 |
| AGB050 | *Hipposideros abae* | *Trypanosoma* sp. A | PV790212 |
| AGB055 | *Hipposideros abae* | *Trypanosoma* sp. A | PV790211 |
| AGB074 | *Hipposideros abae* | *Trypanosoma* sp. A | PV790213 |
| ENE003 | *Hipposideros abae* | Low-quality sequence (closest BLAST match: *Trypanosoma* sp.) | - |
| AGB066 | *Hipposideros jonesi* | Low-quality sequence (closest BLAST match: *Trypanosoma* sp.) | - |
| AGB031 | *Hipposideros ruber* | *Trypanosoma* sp. A | PV790210 |
| AGB032 | *Hipposideros ruber* | *T.* *livingstonei/ T.* cf. *livingstonei* | PV790223 |
| AGB004 | *Hipposideros ruber* | *Trypanosoma* sp. A | PV790215 |
| AGB008 | *Hipposideros ruber* | Low-quality sequence (closest BLAST match: *Trypanosoma* sp.) | - |
| ADA001 | *Mops condylurus* | *Trypanosoma* sp. A | [PV790209](https://www.ncbi.nlm.nih.gov/nucleotide/PP320488.1?report=genbank&log$=nucltop&blast_rank=1&RID=J9KSPAUT013) |
| ADA005 | *Mops condylurus* | *Trypanosoma* sp. A | [PV790217](https://www.ncbi.nlm.nih.gov/nucleotide/OR462731.1?report=genbank&log$=nucltop&blast_rank=1&RID=J9MB85N8013) |
| AGB061 | *Rhinolophus landeri* | *Trypanosoma* sp. A | PV790221 |
| AWH011 | *Hipposideros ruber* | Low-quality sequence (closest BLAST match: *Trypanosoma* sp.) | - |
| NSK010 | *Rhinolophus landeri* | *T.* *livingstonei/ T.* cf. *livingstonei* | PV790224 |
| NGW006 | *Rousettus aegyptiacus* | *Trypanosoma* sp. A | PV790216 |
| Nyct2 | *Hipposideros abae* | Cyclopodia greeffi | PV791354 |
| Nyct3 | *Hipposideros ruber* | Cyclopodia greeffi | PV791355 |
| Nyct4 | *Rousettus aegyptiacus* | Cyclopodia greeffi | PV791356 |
| Nyct5 | *Hipposideros ruber* | *Eucampsipoda africana* | PV791357 |
| Nyct6 | *Hipposideros abae* | *Eucampsipoda africana* | PV791358 |
| Nyct7 | *Hipposideros abae* | *Eucampsipoda africana* | PV791359 |
| Str3 | *Rhinolophus landeri* | Streblidae sp. | PV791360 |
| Str4 | *Rhinolophus alcyone* | Streblidae sp. | PV791361 |
| MT3 | *Hipposideros abae* | *Spinturnix* sp. | PX125989 |

- = sequence of low quality, but highest BLASTn identity with trypanosome sequences
