# Supplementary material for: Cave-dwelling bats as hosts of Trypanosoma livingstonei: Evidence from peri-domestic roosts in southeastern Nigeria
Source: PLoS One. 2026 Aug 12;21(8):e0355840. doi: 10.1371/journal.pone.0355840 (PMC13465849; doi:10.1371/journal.pone.0355840)
Supplement: Supplementary file 6 [file pone.0355840.s005.docx]

Inclusivity in global research

**Ethical considerations, permits and authorship**

*This section is applicable to all research types.*

Provide details as to who granted permissions and/or consent for the study to take place in the Methods section of your manuscript. This should include the names of **all** ethics boards, governmental organizations, community leaders or other bodies that provided approval for the study. If individuals provided approval refer to these people by their role or title but do not list their name(s).

**Reported on page number: 5**

Ethical approval for this study was obtained from the Ethics and Biosafety Committee, Faculty of Biological Sciences, University of Nigeria, Nsukka (approval number: UNN/FBS/EC/1014). Fieldwork and sample collection were conducted with permission from the relevant institutional and regulatory authorities in Nigeria, including the State Ministries of Health and Environment. Where required, access to sampling sites was granted with the approval and support of local community leadership. All bat capture, handling, and sampling procedures followed the guidelines of the American Society of Mammalogists for the use of wild mammals in research, and all efforts were made to minimize stress and harm to the animals. A subset of individuals (14/57) was retained as voucher specimens to allow for re-identification; these individuals were euthanized using isoflurane and preserved in ethanol and are deposited in a Nigerian research collection (Afrotropical Biodiversity Center, permanent field station and collections supported by SMACON, located in Cross River State), ensuring local access to material and supporting future research and capacity building. Fruit bats were provided with a sugar solution prior to release, whereas no supplemental feeding was provided to insectivorous species.

If there were any deviations from the study protocol after approval was obtained please provide details of these changes in the Methods section of your manuscript.

**Reported on page number: [Not applicable (no deviations occurred]**
No deviations from the approved study protocol occurred after ethical approval was obtained. All procedures were conducted in full accordance with the originally approved protocol.

Did this study involve local collaborators that are residents of the country where the research was conducted or members of the community studied? If you do not have any authors from said communities, please provide an explanation for this below.

Everyone listed as an author should meet PLOS’ criteria for authorship and all individuals who meet these criteria should be included in the author byline, rather than the acknowledgements. For further information please see the journal’s Authorship Policy.

Yes. **This study was led by Nigerian researchers** and involved multiple local collaborators who are residents of Nigeria, where the research was conducted. In total, six Nigerian authors contributed to and/or supervised key aspects of the study, including study design, fieldwork, sample collection, and DNA extraction, which was carried out locally in Nigeria. These contributors are included as authors (including the first author) on the manuscript. The study also included three international collaborators, one of whom contributed in a supervisory role, while the others provided specialized expertise in molecular and phylogenetic analyses of blood parasites. Their contributions supported downstream laboratory analyses and data interpretation.

**Human subjects research (e.g. health research, medical research, cross-cultural psychology)**

Did you obtain written informed consent from a representative of the local community or region before the research took place? How did you establish who speaks for the community? Details of written informed consent obtained from study participants should be reported separately in the Methods section of your manuscript.

Not applicable. This study did not involve human participants or human subjects research. The research focused exclusively on wildlife (bats) and associated parasites.

How did members of the local community provide input on the aims of the research investigation, its methodology, and its anticipated outcome(s)?

N/A

When engaging with the local community, how did you ensure that the informed consent documents and other materials could be understood by local stakeholders?

N/A

Will the findings of the research be made available in an understandable format to stakeholders in the community where the study was conducted (e.g. via a presentation, summary report, copies of publications, etc.)? Please provide details of how this will be achieved.

N/A

**Non-human subjects research using specimens/ animals collected as part of the study, or those housed in archival collections. Examples include archaeology, paleontology, botany and zoology.**

Did the permission you obtained from a local authority to perform the study include an agreement on access to outputs and benefit sharing? This may include procedures to enable fair distribution of the benefits and resources arising from the research performed. Please include any details of Prior Informed Consent and Benefit Sharing Agreements obtained. These may be required by field-specific regulations, for example the Convention on Biological Diversity (CBD) and the associated Nagoya Protocol.

Permission for the study was obtained from the Ethics and Biosafety Committee, Faculty of Biological Sciences, University of Nigeria, Nsukka (approval number: UNN/FBS/EC/1014, issued 30 October 2019). This approval covered field sampling, specimen handling, and subsequent laboratory analyses.

The transfer of extracted DNA samples for molecular analysis was conducted under a formal Material Transfer Agreement (MTA) between Humboldt University Berlin and the University of Nigeria, Nsukka, signed on 16 July 2024. This agreement defined the terms of sample use, data access, and collaboration between the institutions. No separate, formal Access and Benefit-Sharing (ABS) agreement or Prior Informed Consent (PIC) framework under the Nagoya Protocol was established beyond the institutional approvals and the MTA. However, the study was conducted as an equitable collaboration, with local researchers leading the study and included as first and co-authors, ensuring shared access to research outputs and benefits arising from the work. Voucher specimens generated in this study are deposited in a Nigerian research collection (the Afrotropical Biodiversity Center), ensuring local access to material and supporting future research and capacity building.

If the material used in your study was imported, please A) provide the year it was imported and B) indicate whether permits were obtained to import/export the materials used, C) provide details of any permits obtained. If this information is not available, please indicate this.

Yes. Extracted DNA samples generated in Nigeria were transferred to Germany in 2024 for molecular analysis. The transferred material consisted solely of extracted DNA and not whole biological specimens.

The samples were derived from arthropod and bat species that are not listed as threatened, and to our knowledge, no specific export or import permits were required for the transfer of this type of material.

The transfer was conducted under a formal Material Transfer Agreement (MTA) between Humboldt University Berlin and the University of Nigeria, Nsukka, signed on 16 July 2024. This agreement governed the transfer, use, and handling of the material in accordance with institutional and applicable regulatory requirements. The extracted DNA was fully utilized during the course of the analyses, and no material remains for return or further transfer.

If you used archival specimens, please state how the material used in your study was acquired by the institute it is held in and provide details of any permits obtained for the original excavations/ sample collection. If this information is not available, please indicate this.

Not applicable. All specimens and samples used in this study were collected prospectively by the research team in Nigeria as part of this study and were not obtained from archival or museum collections.

How was the potential cultural significance of the materials collected in your study to local communities considered in your research design? Were Indigenous peoples and/or local researchers and institutions involved with archaeological excavations / collection of specimens? If so, please provide a description of their involvement.

The study focused on the collection of bat blood samples for parasitological and molecular analyses, which are not known to have specific cultural or archaeological significance to local communities.

Local researchers and institutions in Nigeria were actively involved in all stages of the study, including study design, fieldwork, and specimen collection. Field sampling was conducted with the approval and support of local community leadership where required, ensuring that collection activities were carried out in a culturally appropriate and locally accepted manner.

No Indigenous cultural materials or traditional knowledge were directly targeted or collected as part of this study.

If your manuscript includes photographs of human remains please indicate whether authors obtained permission from descendants or affiliated cultural communities to do so.

N/A
